# Leishmania enriettii visceralises in the trachea, lungs, and spleen of Cavia porcellus

**DOI:** 10.1590/0074-02760220065

**Published:** 2022-08-01

**Authors:** Ednéia Venâncio Alves-Sobrinho, Lucélia de Jesus Pinheiro, Larissa Ferreira Paranaíba, Igor Campos Fontes, Patrícia Martins Parreiras, Nelder Figueiredo Gontijo, Wagner Luiz Tafuri, Márcia Dalastra Laurenti, Rodrigo Pedro Soares

**Affiliations:** 1 Universidade Federal de Minas Gerais, Instituto de Ciências Biológicas, Departamento de Parasitologia, Belo Horizonte, MG, Brasil; 2 Universidade Federal de Minas Gerais, Instituto de Ciências Biológicas, Departamento de Patologia Geral, Belo Horizonte, MG, Brasil; 3 Fundação Oswaldo Cruz-Fiocruz, Instituto René Rachou, Belo Horizonte, MG, Brasil; 4 Universidade de São Paulo, Faculdade de Medicina, Laboratório de Patologia de Moléstias Infecciosas, São Paulo, SP, Brasil

**Keywords:** Leishmania enriettii, Cavia porcellus, subgenus *Mundinia*, visceralisation, histopathology, host-parasite interaction

## Abstract

**BACKGROUND:**

*Leishmania* (*Mundinia*) *enriettii* is a species commonly found in the guinea pig, *Cavia porcellus*. Although it is a dermotropic species, there is still an uncertainty regarding its ability to visceralise during *Leishmania* life cycle.

**OBJECTIVE:**

Here, we investigated the ability of *L. enriettii* (strain L88) to visceralise in lungs, trachea, spleen, and liver of *C. porcellus*, its natural vertebrate host.

**METHODS:**

Animals were infected sub-cutaneously in the nose and followed for 12 weeks using histological (hematoxilin-eosin) and molecular tools (polymerase chain reaction-restriction fragment length polymorphism - PCR-RFLP). To isolate parasite from *C. porcellus*, animals were experimentally infected for viscera removal and PCR typing targeting *hsp70* gene.

**FINDINGS:**

Histological analysis revealed intense and diffuse inflammation with the presence of amastigotes in the trachea, lung, and spleen up to 12 weeks post-infection (PI). Molecular analysis of paraffin-embedded tissues detected parasite DNA in the trachea and spleen between the 4th and 8th weeks PI. At the 12th PI, no parasite DNA was detected in any of the organs. To confirm that the spleen could serve as a temporary site for *L. enriettii*, we performed additional *in vivo* experiments. During 6th week PI, the parasite was isolated from the spleen confirming previous histopathological and PCR observations.

**MAIN CONCLUSION:**

*Leishmania enriettii* (strain L88) was able to visceralise in the trachea, lung, and spleen of *C. porcellus*.

*Leishmania enriettii* is a parasite belonging to the subgenus *Mundinia*,^1^ ^,^ ^2^ whose vertebrate host is the guinea pig, *Cavia porcellus*. Other members of this subgenus include *Leishmania* (*M.*) *macropodum*,^3^ *Leishmania* (*M.*) *martiniquensis*,^4^ *Leishmania* (*M.*) *orientalis* ^5^ and *Leishmania* (*Mundinia*) sp. (Ghana isolate).^6^ Several whole genome sequencing studies that have been published aimed to understand taxonomic relationships among the *Mundinia*, *Leishmania* and *Viannia* subgenera.^7^ ^,^ ^8^ ^,^ ^9^ However, there is still a substantial uncertainty regarding some aspects of these parasites biology.

An interesting feature of the members of this subgenus is their ability to infect non-phlebotomine vectors, such as ceratopogonids (*Culicoides* spp.).^10^ ^,^ ^11^ Recently, transmission by those vectors has been experimentally demonstrated.^12^ In Brazil, a proven vector for *L. enriettii* is yet to be determined, although *Lutzomyia monticula* has been suggested.^13^ A recent study isolated several *L. enriettii* strains in the State of Paraná, Brazil. It did not detect the parasite in the wild reservoirs reinforcing the role of *C. porcellus* as a the main, if not the only, domestic host.^14^

Several research groups have been studied *L. enriettii* to understand its biology and some aspects of host-parasite interaction.^15^ ^,^ ^16^ A distinguished feature of this species (unlike other *Mundinia* members) is its ability to specifically infect guinea pigs.^17^ In *C. porcellus*, *L. enriettii* causes a severe ulcerated cutaneous lesion (CL) that heals over time. For this reason, *C. porcellus* was used as a model for CL for many years.^18^ ^,^ ^19^ Experimental infection of guinea pigs by *L. enriettii* did not use salivary glands extracts (SGE) until recent biological and immunopathological studies.^20^ ^,^ ^21^ *Lutzomyia longipalpis* SGEs modulated the infection by preventing the attraction of monocytes and CD163 macrophages throughout the course of infection. After 12 weeks post-infection (PI) regardless the use of SGEs, the CL lesions healed spontaneously. Early and recent reports^14^ ^,^ ^18^ ^,^ ^21^ ^,^ ^22^ ^,^ ^23^ already demonstrated the ability of *L. enriettii* to visceralise in guinea pigs after several inoculation routes. It was, however, unknown if this phenomenon is permanent or transient requiring fast transmission to vector(s).

Over the 75 years since the discovery of *L. enriettii*, there are several aspects of infection that still need to be better elucidated. In our previous study, we reported the proinflammatory features of *L. enriettii* infection in the skin.^20^ After the removal of the skin for immunopathological studies, we also collected trachea, lungs, liver, and spleen in the same PI time intervals. Here, as part of a wider study of *L. enriettii*, we provide a more detailed study on the immunopathological aspects in different organs using molecular and histological approaches in *C. porcellus* viscera.

## MATERIALS AND METHODS

*Histology evaluations* - Trachea, lung, liver, and spleen were recovered from our previous study.^21^ In that paper, animals were subcutaneously infected with 1 × 10^5^ promastigotes of *L. enriettii* reference strain (MCAV/BR/1945/L88) in 0.1 mL of phosphate-buffered saline (PBS) supplemented with 1/2 salivary gland of *L. longipalpis*. Animals (n = 12) were followed for 12 weeks and euthanised with an overdose of ketamine and xylazine (500 mg/kg and 100 mg/kg) at weeks four (n = 3), eight (n = 3), and 12 (n = 3). Negative controls included three animals euthanised at weeks four, eight, and 12. Tissue fragments were fixed in a 10% buffered formalin solution, pH 7.2 for 48 hours. Paraffin blocks were cut in a microtome (3-4 μm) and mounted on slides prior to routine histological analysis (hematoxylin-eosin - HE). Histological slides were qualitatively analysed under light microscopy as previously reported.^21^

*Parasite detection in paraffin tissues* - Paraffin-embedded tissue samples (trachea, lung, spleen, and liver) were subjected to DNA extraction^24^ prior to amplification of *hsp70* gene (~1,300 bp).^25^ The fragment was visualised in 4% agarose gel.

*In vivo experiments* - For visceralisation confirmation, six experimental animals were divided in two groups including: (1) two negative controls inoculated with (PBS+SGE) and (2) four animals infected with *L. enriettii*. Guinea pigs were infected intradermally with 10^5^ *L. enriettii* promastigotes in PBS + SGE from *L. longipalpis* as previously reported.^20^ After six weeks PI, tissue fragments were recovered for parasite isolation.

*Parasite recovery and typing* - Tissue fragments from trachea, lungs, liver, and spleen were seeded in NNN-Schneider’s medium and incubated at 26**º**C. Cultures were followed until appearance of promastigote forms. Positive cultures were expanded for DNA extraction and polymerase chain reaction (PCR) typing using *hsp70* gene as above. The ~1,300-bp fragment was subjected to digestion with *Hae*III (New England) and restriction profiles were analysed in a 4% agarose gel.^25^

*Ethical considerations* - This work was approved by the Ethics Committee for Animal Use (CEUA), Oswaldo Cruz Foundation (FIOCRUZ) (LW-24/19). Young male *C. porcellus* were bred according to the International Standards for the Breeding and Use of Laboratory Animals,^26^ obtained from the Institute of Science and Technology in Biomodels (ICTB/Fiocruz).

## RESULTS

**Fig. 1:**
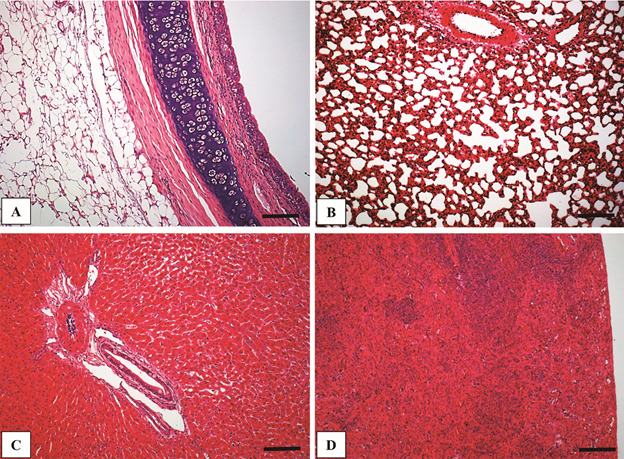
panoramic view of trachea (A), lung (B), liver (C) and spleen (D) of uninfected *Cavia porcellus* at 8th weeks post-infection (PI) (hematoxylin-eosin - HE) (Bar = 32 µm).

**Fig. 2:**
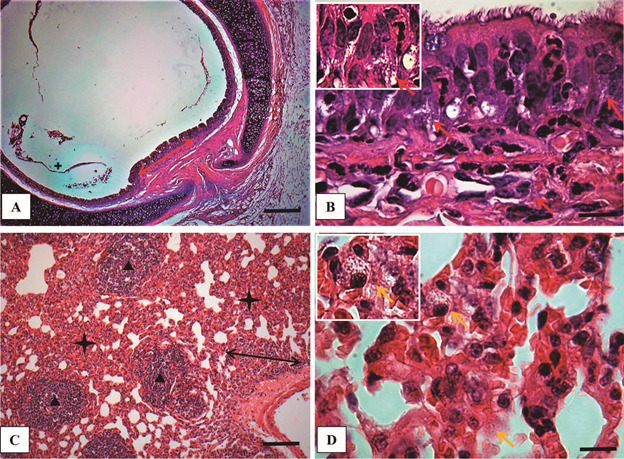
histological evaluation of trachea (A - B) and lung (C -D) of *Cavia porcellus* experimentally infected with *Leishmania enriettii* at 8th weeks post-infection (PI) (hematoxylin-eosin - HE). (A) Panoramic view of the trachea showing a diffuse chronic inflammatory reaction (red arrow) (Bar = 64 mm); (B) High magnification of (A) showing the chronic exudate of mononuclear cells with parasitised macrophages (red arrow) (Bar = 16 µm). (C) Low magnification of pulmonary parenchyma showing an intense chronic inflammatory reaction (black stars), an increase in the thickness of the alveolar wall (double headed arrow) and, granulomatous reaction formation (black triangles) (Bar = 32 μm). (D) High magnification of pulmonary alveolar wall showing a chronic inflammatory exudate with macrophages containing *L. enriettii* amastigotes (yellow arrows) (Bar = 16 μm). Higher magnification on the upper left corner in (B) and (D) showing an infected macrophage.

**Fig. 3:**
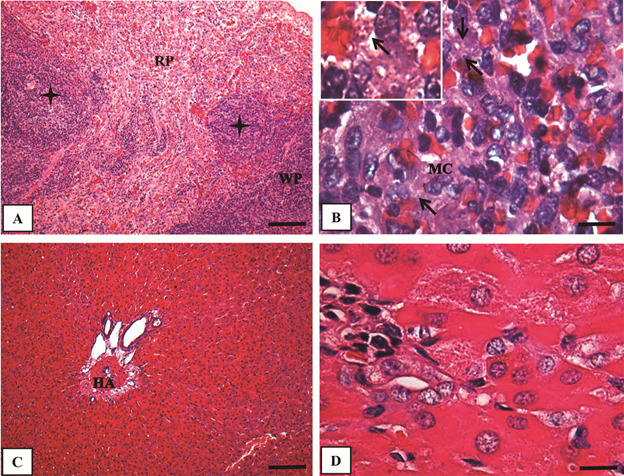
histological evaluation of spleen (A - B) and liver (C - D) of *Cavia porcellus* experimentally infected with *Leishmania enriettii* at 8th weeks post-infection (PI) (hematoxylin-eosin - HE). (A) Panoramic view of the spleen showing the red pulp with a dense and diffuse inflammatory infiltrate composed of mononuclear cells and granuloma formation (black stars) (Bar = 64 μm). (B) High magnification of (A) with macrophages (MC) containing *L. enriettii* amastigotes (thin arrow) (Bar = 16 μm). (C) Panoramic view of the liver with normal hepatocyte cords and branch of the hepatic artery (Bar = 64 μm). (D) High magnification of (C) showing regular cells (Bar = 16 μm). Higher magnification on the upper left corner in (B) showing an infected macrophage. RP: red pulp; WP: white pulp; HA: hepatic artery; MC: macrophage.

**Fig. 4:**
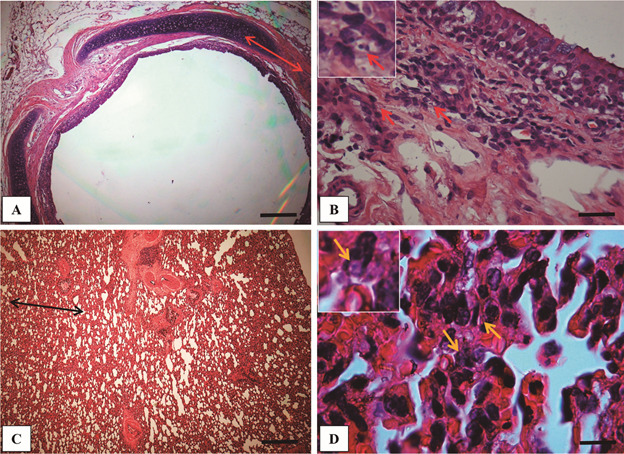
histological evaluation of trachea (A -B) and lung (C - D) of *Cavia porcellus* experimentally infected with *Leishmania enriettii* at 12th weeks post-infection (PI) (hematoxylin-eosin - HE). Panoramic view of the trachea still showing a chronic diffuse reaction (red arrow) (Bar = 64 μm); (B) Large magnification of (A) showing the exudate from mononuclear cells with parasitised macrophages (red arrow) (Bar = 16 μm). (C) Low magnification of pulmonary parenchyma still showing an intense chronic inflammatory reaction, increase in the thickness of the alveolar wall (double headed arrow) and resolution of granulomatous reaction (Bar = 32 μm). (D) Higher magnification of (C) showing alveolar walls with an inflammatory exudate and macrophages (yellow arrows) (Bar = 16 μm).

**Fig. 5:**
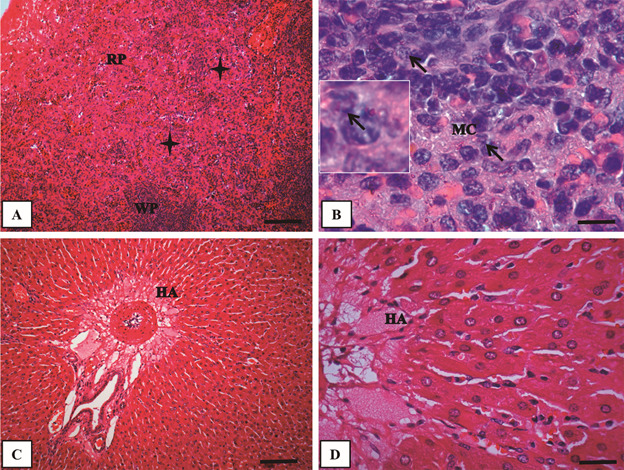
histological evaluation of the spleen (A - B) and liver (C - D) of *Cavia porcellus* experimentally infected with *Leishmania enriettii* at 12th weeks post-infection (PI) (hematoxylin-eosin - HE). (A) Panoramic view of the spleen showing the red pulp with a dense and diffuse inflammatory infiltrate composed of mononuclear cells and granuloma formation (black stars) (Bar = 64 μm). (B) High magnification of (A) with macrophages containing *L. enriettii* amastigotes (thin arrow) (Bar = 16 μm). (C) Panoramic view of the liver with normal hepatocyte cords and hepatic artery branch (Bar = 64 μm). (D) High magnification of (C) showing regular cells (Bar = 16 μm). RP: red pulp; WP: white pulp; HA: hepatic artery; MC: macrophage

*Histopathological evaluation* - Histopathological changes in the trachea, lung, spleen, and liver of infected animals were analysed at the 4th, 8th, and 12th weeks PI using HE. Negative controls with uninfected animals did not show any histological alterations (Fig. 1A-D). Based on our previous work, the cutaneous lesions peak at 6th-8th weeks PI.^21^ For this reason, we chose representative images at 8th and 12th weeks PI (Figs 2-5). In the mucosa and submucosal region, trachea showed intense and diffuse mononuclear inflammatory infiltrate with amastigotes inside macrophages (Fig. 2A-B). In the lungs, the main lesion observed was a diffuse and intense chronic inflammatory reaction with granuloma formation characterising an interstitial pneumonitis (Fig. 2C). In fact, the alveolar wall was thickened because of the presence of the chronic exudates of plasma cells, lymphocytes, and several parasitised macrophages with numerous amastigotes of *L. enriettii* (Fig. 2D).

In the spleen, the histological alterations included thickening and inflammation of the capsule and, hyperplasia/hypertrophy of the white and red pulp (Fig. 3A). The hyperplasia and hypertrophy were characterised by numerous enlarged macrophages usually parasitised with *L. enriettii* amastigotes. In parallel, the sinusoid vessels of the red pulp were always enlarged and congested (Fig. 3B). Histopathological alterations and amastigote forms were not documented in the liver (Fig. 3C-D).

At 12 weeks PI, most of the histopathological features in the organs persisted. Trachea, lungs, and spleen still had inflammation with productive proinflammatory infiltrate and parasite presence, whereas liver did not change (Figs 4-5). However, an interesting feature was shown in the lungs (Fig. 4B). Although pneumonitis was still evident, a decrease in the granulomas was noticed.

**Fig. 6:**
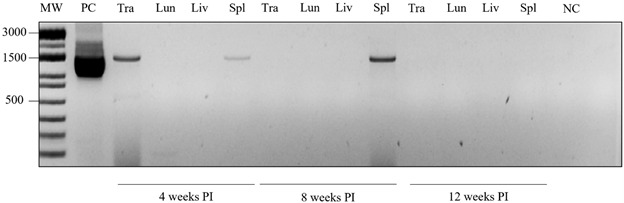
DNA detection in *Cavia porcellus* viscera infected with *Leishmania enriettii* targeting *hsp70* gene at different time intervals. MW: molecular weight; PC: positive control; Tra: trachea; Lun: lung; Liv: liver; SPL: spleen; NC: negative control.

*Leishmania enrietti DNA detection in C. porcellus viscera* - The expected ~1,300-bp fragment was detected in the positive control (PC) (L88 strain of *L. enriettii*), trachea (Tra) (4th week PI) and spleen (4th and 8th weeks PI). At 12 weeks PI, no DNA was detected in the organs (Fig. 6).

**Fig. 7:**
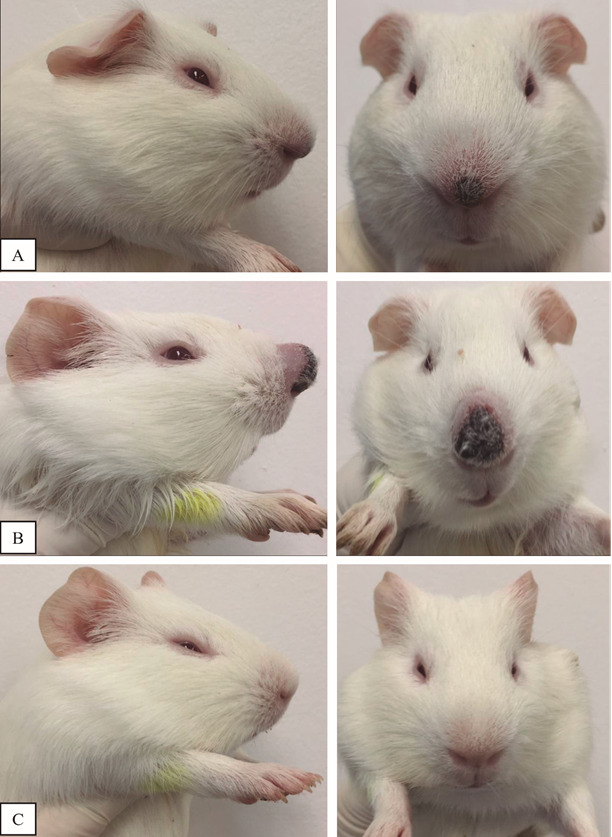
experimental infection of *Cavia porcellus* by *Leishmania enriettii*. Lesion development at 4th (A) and 6th weeks (B) post-infection (PI) showing typical nodular ulcerated lesion with alopecia. (C) uninfected animals (negative controls).

*Experimental infection of C. porcellus with L. enriettii* - Confirming our previous findings,^20^ ^,^ ^21^ L88 strain successfully infected *C. porcellus* showing cutaneous lesion development and swelling of the nasal plane at 4th and 6th weeks PI (Fig. 7A-B). Macroscopically, the cutaneous lesion showed an expected nodular protuberance with alopecia and ulceration common to *L. enriettii*. As expected, control animals did not develop any cutaneous lesions (Fig. 7C).

**Fig. 8:**
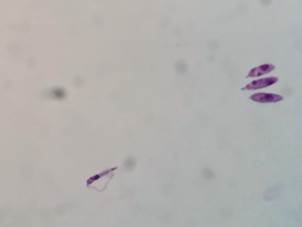
*Leishmania enriettii* (L88 strain) isolated from *Cavia porcellus* spleen after 6th week post-infection (PI). Magnification (1000X).

**Fig. 9:**
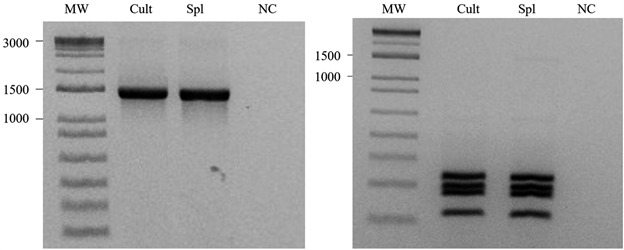
molecular identification of *Leishmania enriettii* during experimental infection in *Cavia porcellus*. (A) amplification of *hsp70* fragment and (B), digestion with *Hae*III. MW: molecular weight; Cult: parasites from culture; Spl: parasites isolated from spleen; NC: negative control.

*Parasitological and molecular confirmation of L. enriettii visceralisation in C. porcellus* - After 18 days, spleen-derived cultures revealed presence of promastigotes from one animal (Fig. 8). As expected, the ~1,300 bp fragment was amplified from both samples (Fig. 9A). After treatment with *Hae*III, their profiles were the same (Fig. 9B).

## DISCUSSION

Histopathological and molecular studies involving species from the subgenus *Mundinia* have so far been restricted to the dermis region.^3^ ^,^ ^17^ ^,^ ^20^ ^,^ ^21^ However, an unknown aspect of the infection outcome is its progression to the parasitological cure or latency. In this work we carried out a more detailed analyses in the viscera of *C. porcellus* infected with *L. enriettii* to corroborate findings of its visceralisation. In the previous papers, the ability of *L. enriettii* to infect several organs, including spleen, liver, adrenal glands, bone marrow, lymphatic ganglia and even blood, was reported.^14^ ^,^ ^18^ ^,^ ^19^ ^,^ ^23^ Here, we reported for the first time the infection in trachea and lungs. By the time abovementioned papers were published, molecular tools were not available, and the techniques were mainly restricted to parasitological and microscopical methods.

Consistent with previous observations, we detected amastigote forms of *L. enriettii* in trachea, lung, and spleen. Our histopathological findings showed intense inflammatory processes in those organs, except for liver that did not show any changes. Lungs were the most affected organs, where the inflammatory profile was more exacerbated with the presence of numerous granulomas and interstitial pneumonitis. However, they disappeared at 12th weeks PI, suggesting a healing process. This atypical alteration in this organ (granulomes) was similar to those caused by typical viscerotropic species in hamsters’ liver.^27^ ^,^ ^28^ This is different from dogs naturally infected with *L. infantum*, where only chronic interstitial pneumonitis with notable thickening of the interalveolar septa were noted.^29^ Although there are evident pneumonia associated with granuloma and pyogranulomas, the findings of amastigotes in dogs lungs are very scarce. This is different from guinea pigs, where it was possible to visualise amastigotes in several areas. An interesting feature of this work is the apparent absence of amastigotes and/or pro-inflammatory processes in the liver. On the other hand, those were detected in the spleen during the histological assessment with noticeable intracellular amastigote forms in the parenchyma, especially inside macrophages. Altogether, our data indicate that *L. enriettii* can visceralise and cause immunopathological events in trachea, lungs, and spleen up to 12 weeks PI. While our previous paper^21^ demonstrated that the infection in the skin was completely cured at 12th PI, it remained active in the viscera.

To confirm this, paraffin-embedded tissues were assayed for the presence of parasite DNA using molecular tools. Recently, *hsp70* gene has been successfully used as a tool for discriminating *L. martiniquensis* and *L. orientalis*.^30^ Here, we amplified the specific fragment of this target (~1,300-bp) from DNA extracted from paraffin-embedded tissues.^25^ This has allowed us to trace movement of *L. enriettii* from trachea to spleen in the first 8 weeks PI. After two months, it disappears from the trachea and increases its presence in the spleen. No parasite DNA was found at 12 weeks PI suggesting a possible clearance of the parasite in the organs. However, this result is conflicting with the histopathological findings that showed amastigote forms in the tissues during this period. This also does not correlate with what occurs in the skin of the same animals, whose lesions reached their peak at 6 weeks PI and are completely healed at 12 weeks.^20^ ^,^ ^21^ Confirming our previous histological findings, no DNA was found in the liver in all time points analysed. However, it was also not detected in the lungs by PCR, where amastigotes and a severe pro-inflammatory milieu were seen, implying sensitivity limits of PCR detection. Interestingly, our histopathological analysis showed the presence of tracheal, lung and spleen amastigotes in all analysed weeks, including the 12th week PI. Although those techniques may be complementary, inconsistencies are expected since DNA extraction from paraffin-embedded tissues is laborious and may affect DNA quality, especially at 12th weeks PI, where fewer parasites were seen. This is different from previous studies, where detection of parasite DNA in paraffin blocks with virtual absence of amastigotes was achieved.^29^ ^,^ ^31^ This reinforces the need of using different methods for description of parasitological events in the hosts.

Based on our data, it is likely that *L. enriettii* can reach the viscera, cause severe histopathological alterations that decrease, but not completely disappear, for example, in the skin 8-12-weeks PI. It is interesting to notice that under our controlled experimental conditions the animals were apparently active and did not have any clinical signs.^20^ ^,^ ^21^ However, this is different from previous studies with infected field-collected guinea pigs, where the severity of the lesions seemed to have an impact in those animals.^14^ ^,^ ^22^ We suggest that in nature a higher severity of the lesions may provide more infective blood meal to the vector. However, a proven vector for *L. enriettii* is yet to be determined.^15^

Leishmaniasis comprises a wide spectrum of clinical manifestations including diseases caused by dermotropic and viscerotropic species. Depending on the region, intraspecies tropism variations may occur. For example, *Leishmania infantum* (viscerotropic) can cause cutaneous lesions in Honduras, Central America.^32^ *Leishmania donovani*, another viscerotropic species, can cause unusual dermal lesions after therapeutic failure known as Post-kala-azar dermal Leishmaniasis (PKDL).^33^ The opposite is also true for *Leishmania amazonensis* (dermotropic). It was isolated from lymph nodes from dogs showing symptoms of canine visceral leishmaniasis, similar to that caused by *L. infantum*,^34^ and from rodents’ viscera.^35^ Finally, *L. martiniquensis*, another member of the *Mundinia* subgenus, causes not only LV, but also CL in HIV-positive patients.^36^ Consistent with those observations, *L. enriettii* showed a dual profile being able to cause dermotropic and viscerotropic lesions under our experimental conditions in its main host *C. porcellus*.

To confirm our previous histological and molecular findings, next we performed an *in vivo* infection targeting the spleen as a possible source for parasite isolation and observed the same pattern of lesion development as reported elsewhere at four-eight weeks PI.^20^ ^,^ ^21^ After euthanasia, we failed to isolate parasites from trachea, lungs, and liver fragments after six weeks PI. After 18 days, we succeed in detecting promastigotes in only one spleen fragment from one animal. After performing polymerase chain reaction-restriction fragment length polymorphism (PCR-RFLP), we confirmed that cultured promastigotes used for animal infection had the same profile as those, recovered from the spleen. This finding confirms that live parasites can be isolated from spleen.

*Leishmania enriettii* (strain L88) visceralised in *C. porcellus* causing severe lesions in lungs, trachea, and spleen. Despite the clinical cure of the skin lesion, the parasite was not eliminated from the tissues during the 12 weeks PI as judged by the histological studies. In contrast to the infected field-collected guinea pigs, *L. enriettii* under laboratory conditions did not cause any clinical sign to them. This finding is of epidemiological importance, since those animals may be potential sources for vector infection facilitating parasite maintenance and spreading in the environment. This phenomenon warrants further investigation to address the role of apparently cutaneous healed animals in *L. enriettii* transmission.
